# In vitro hair follicle growth model for drug testing

**DOI:** 10.1038/s41598-023-31842-y

**Published:** 2023-03-24

**Authors:** Tatsuto Kageyama, Hikaru Miyata, Jieun Seo, Ayaka Nanmo, Junji Fukuda

**Affiliations:** 1 Faculty of Engineering, Yokohama National University, 79-5 Tokiwadai, Hodogaya-ku, Yokohama, Kanagawa 240-8501 Japan; 2 Kanagawa Institute of Industrial Science and Technology, 3-2-1 Sakado, Takatsu-ku, Kawasaki, Kanagawa 213-0012 Japan; 3 Japan Science and Technology Agency (JST)-PRESTO, 4-1-8 Honcho, Kawaguchi, Saitama, 332-0012 Japan

**Keywords:** Biomaterials, Tissue engineering

## Abstract

In vitro models of human hair follicle-like tissue could be fundamental tools to better understand hair follicle morphogenesis and hair drug screening*.* During prenatal development and postnatal cyclic hair regeneration, hair follicle morphogenesis is triggered by reciprocal interactions and the organization of the epithelial and mesenchymal cell populations. Given this mechanism, we developed an approach to induce hair peg-like sprouting in organoid cultures composed of epithelial and mesenchymal cells. Human fetal/adult epithelial and mesenchymal cells were cultured in a medium supplemented with a low concentration of either Matrigel or collagen I. These extracellular matrices significantly enhanced the self-organization capabilities of the epithelial and mesenchymal cells, resulting in spherical aggregation and subsequent hair peg-like sprouting. The length of the hair peg sprouting and associated gene expression significantly increased in the presence of a well-known hair drug, minoxidil. This approach may be beneficial for testing hair growth-promoting drug candidates.

## Introduction

Hair loss is caused by various factors, including genetics, aging, hormonal imbalances, autoimmune reactions, stress, and anti-cancer medications^1,2^. Due to the influence of hairstyles on one’s appearance, there is a high demand for hair loss treatments. The leading treatment for hair loss is pharmacotherapy, but it still has some limitations, such as poor outcomes and side effects^3^. Hence, developing more effective and less harmful drugs is important in the cosmetics and pharmaceutical industries. In many cases, the primary step in drug discovery is to screen compounds using cells in a conventional culture dish^4^. The effects of lead compounds are evaluated through the proliferation of epithelial lineage cells and trichogenic gene expression in a culture dish, but the results obtained with this approach do not often reflect the effects in vivo^5^. Thus, a culture system that more closely represents in vivo tissue responses and morphogenesis is desired for accurate drug screening.

To replicate in vivo cellular responses, researchers have developed several in vitro culture models considering in vivo microenvironments^5,6^. Dermal papilla (DP) cells are specialized mesenchymal cells that play a crucial role in regulating the growth and cycling of hair follicles^7^. Forming three-dimensional (3D) DP spheroids recapitulating in vivo tissue morphology improved cellular responses against drugs compared to conventional 2D culture^8^. Epithelial and mesenchymal interactions (EMIs) are crucial in hair follicle growth and cycling in vivo. For example, hair follicle morphogenesis is initiated via the formation of hair follicle germs which trigger EMIs^9^. Cyclic hair follicle regeneration is governed by EMIs even after birth^10^. Bioengineered hair follicle germ-like aggregates composed of epithelial and mesenchymal cell aggregates efficiently generated de novo hair follicles upon intracutaneous transplantation into the back skin of nude mice^11–14^. In vitro hair follicle generation was also reported by recapitulating hair follicle microenvironments and EMIs using 3D-printed molds^15^. Although this approach paved a new way to regenerate human hair follicles in vitro, further improvement of the regeneration efficiency of hair follicles may be required for drug testing. A recent study reported hair-bearing skin organoids using human pluripotent stem cells^16^. The skin organoids were composed of a stratified epidermis, fat-rich dermis, pigmented hair follicles with sebaceous glands, and a network of sensory neurons. This skin organoid model enabled researchers to understand the basic developmental biology of hair follicles and their pathophysiology, such as the relationship between SARS-CoV-2 infection and hair loss^17^. However, this approach might be very costly and unsuitable for drug testing since a substantially long culture period (~ 140 days) is required to differentiate pluripotent stem cells into skin tissues, where unexpected cell types and their secreted proteins may also be included.

**Figure 1:**
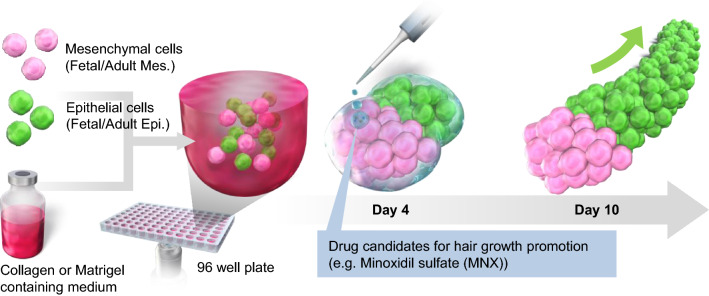
Schematic representation of the preparation of human hair follicle growth models (termed hair follicloids). Hair follicloids are formed through the self-organization of human epithelial and mesenchymal cells, which generate sprouting structures in vitro. Changes in sprouting length were investigated after exposure to hair growth-promoting drug candidates.

We recently developed a culture system to regenerate hair follicles in vitro with mouse embryonic skin-derived cells at very high efficiency (~ 100%) in a short culture period (approximately 6 days)^18^. In this approach, fetal epithelial and mesenchymal cells were seeded in a spheroid culture plate in a culture medium supplemented with Matrigel or collagen I at a considerably low concentration. The cells initially formed aggregates, then spontaneously generated hair follicles through EMIs. In this study (Fig. 1), we examined this approach using human fetal or adult epithelial and mesenchymal cells to develop human hair follicle models. Histological and gene expression analyses were performed to characterize initial/partial hair follicle morphogenesis generated with this approach. Furthermore, we investigated this approach with a hair growth-promoting drug based on hair follicle sprouting. This approach may be promising as a drug testing platform for treating hair loss disorders.

## Results and discussion

### Length and efficacy of hair peg-like sprouting

Hair drugs have been evaluated using cells grown in conventional 2D culture, but drug candidates selected with this approach have sometimes turned out ineffective in vivo. This is probably because cell proliferation and gene expression in non-physiological culture environments are unreliable markers. A cell culture approach that more closely mimics the in vivo microenvironment is needed, and eventual morphological changes, such as hair follicle generation and growth, rather than temporal cellular responses, such as cell proliferation and gene expression in 2D culture, should be used as key indicators. Recently, we developed a biomimetic culture system in which mouse embryonic epithelial and mesenchymal cells formed aggregates and generated hair follicles in vitro^18^. In this organoid culture, de novo hair follicles sprouted in the aggregate with near 100% efficiency. A key approach in culturing hair follicle organoids is to supplement the culture with Matrigel or collagen I at a sufficiently low concentration (2 v/v%) and kept at 4 °C even after cell seeding for at least 30 min so as not to form a hydrogel and prevent cell aggregation, which is critical for EMIs.

**Figure 2:**
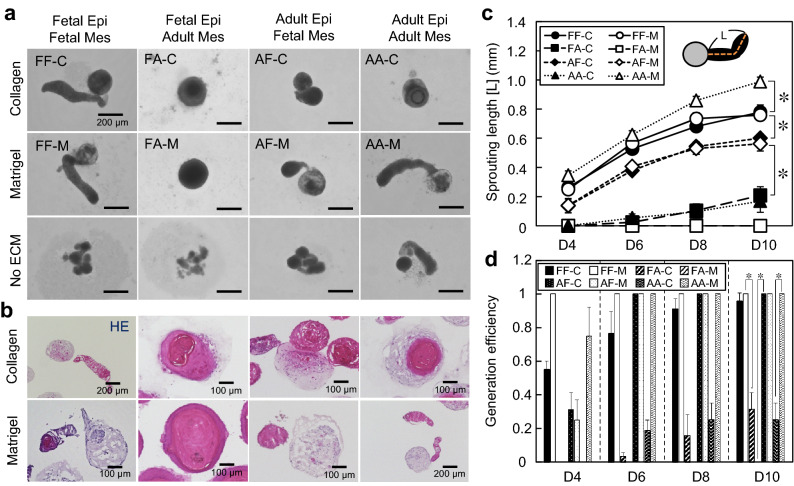
Characterization of human hair follicloids. (**a**) Self-organization of the epithelial and mesenchymal cells in hair follicloids. Hair follicloids were prepared by combining fetal and adult epithelial and mesenchymal cells, supplemented with or without collagen and Matrigel (Table 1). Stereomicroscope images were observed on day ten of culture. (**b**) Cross-sectional view of hair follicloids. On day ten of culture, hair follicloids were sectioned and stained with hematoxylin and eosin. (**c**) Length of the sprouting structures generated from hair follicloids prepared under different conditions (Table 2). The length was measured from the stereomicroscope images taken on days four, six, eight, and ten of culture. (**d**) Generation efficiency of sprouting structures after day ten of culture. The average ratio was analyzed from the results of three independent experiments.

In this study, we investigated human fetal/adult epithelial and mesenchymal cells using our system (see Tables 1 and 2 for the abbreviations of each cell type and the combinations of cell types and matrices, respectively). Under four conditions (FF-C, FF-M, AF-M, AA-M) with a total of eight combinations, the cells initially gathered and aggregated in the U-shaped bottom of a 96-well plate and formed hair peg-like sprouts spontaneously within 4 days of culture, which further elongated in the following 10 days of culture (Fig. 2a, b). The cells grown in a culture medium without collagen and Matrigel supplementation tended to form separate aggregates (Fig. 2a). In the monoculture, epithelial cells formed spherical aggregates but not hair peg-like structures (Supplemental Fig. 1), suggesting that EMIs are closely associated with hair peg-like sprouting formation. The average length of the hair peg-like sprouts and the generation efficiency in FF-M, FF-C, and AA-M were higher than in AF-M and AF-C (Fig. 2c, d). Note that the length of sprouting was determined by measuring the projected length of a curve through the center of the tissue, as shown in the inset in Fig. 2c. The hair peg-like sprouting in AA-M reached ~ 1 mm with a high generation efficiency (~ 100%) at 10 days of culture. The generation efficiency of hair peg-like sprouting in FA-C, FA-M, and AA-C was lower than in AF-M and AF-C.

**Table 1:** Abbreviations for the epithelial and mesenchymal cells.

Cell types	Phenotypes	Donor type	Abbreviations
Epidermal keratinocyte	Epithelial	Fetal	**Fetal Epi**
Hair follicle keratinocyte	Epithelial	Adult	**Adult Epi**
Dermal fibroblast	Mesenchymal	Fetal	**Fetal Mes**
DP cells	Mesenchymal	Adult	**Adult Mes**

**Table 2:** Abbreviations for the experimental conditions.

Epithelial cells	Mesenchymal cells	Supplements	Abbreviations
Fetal Epi	Fetal Mes	2% v/v Matrigel	**FF-M**
Fetal Epi	Fetal Mes	2% v/v Collagen	**FF-C**
Fetal Epi	Fetal Mes	–	**FF-No**
Fetal Epi	Adult Mes	2% v/v Matrigel	**FA-M**
Fetal Epi	Adult Mes	2% v/v Collagen	**FA-C**
Fetal Epi	Adult Mes	–	**FA-No**
Adult Epi	Fetal Mes	2% v/v Matrigel	**AF-M**
Adult Epi	Fetal Mes	2% v/v Collagen	**AF-C**
Adult Epi	Fetal Mes	–	**AF-No**
Adult Epi	Adult Mes	2% v/v Matrigel	**AA-M**
Adult Epi	Adult Mes	2% v/v Collagen	**AA-C**
Adult Epi	Adult Mes	–	**AA-No**

These results were partially unexpected. Murine embryonic cells have a strong ability to generate de novo hair follicles when transplanted into the skin of mice and have been used in research on hair regeneration^19^. However, adult epithelial and mesenchymal cells isolated from hair follicles, especially of human origin, have shown considerably lower or almost no hair-inductive ability^20^. Thus, the feasibility of a new approach has typically been examined using a unique combination of cells, such as human adult epithelial cells and murine fetal mesenchymal cells or murine fetal epithelial cells and human adult mesenchymal cells^21–23^. Considering these facts, it is surprising that AA-M provided the best condition for hair peg-like sprouting among the combinations used in this study (Fig. 2c). We then further investigated whether AA-M could be used to test a hair drug as described below. The mechanisms of the working EMIs, including the signaling pathways involved, will be investigated in more detail in the future using time-course gene expression assays and extensive signal transduction analyses.

### Immunohistochemical analysis of hair peg-like sprouting

**Figure 3:**
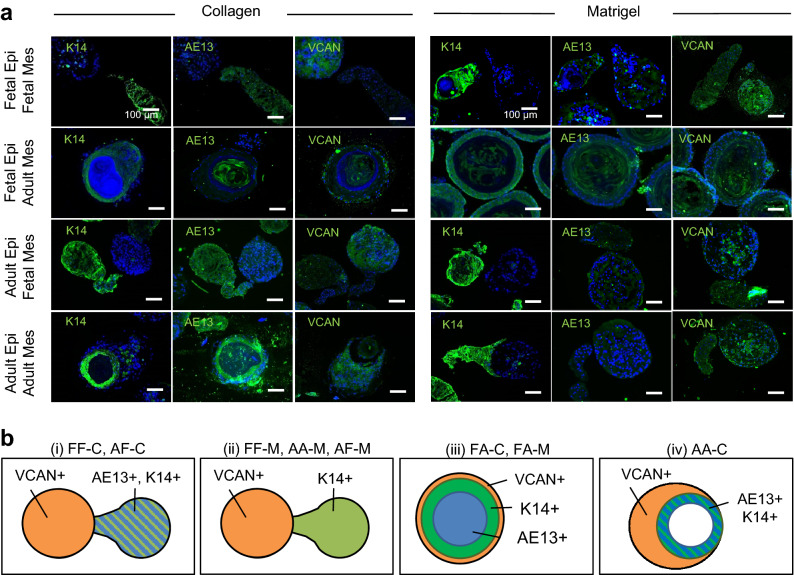
Immunostaining of hair follicloids. (**a**) Histological analysis of hair follicloids. Hair follicles were sectioned and stained with fluorescently labeled antibodies against K14, AE13, and versican. (**b**) Schematics of K14, AE13, and versican expression in hair follicloids.

We characterized the hair peg-like sprouts by staining tissue-specific proteins and stem/progenitor cells at 10 days of culture (Fig. 3a). Immunohistochemical staining revealed that K14 was strongly expressed in the outer root sheath (ORS) in all the sprouting structures. The hair cortex cell marker AE13 was strongly expressed in the sprouting structures in FF-C and AF-C, while a low AE13 expression was observed in FF-M, AA-M, and AF-M. The DP cell marker, versican, was expressed in the mesenchymal aggregates. As depicted in Fig. 3b, the cells in FA-C and FA-M formed three-layered aggregates composed of an AE13^+^ inner core, K14^+^ middle core, and versican^+^ outer shell. The cells in AA-C also formed three-layered aggregates, but the inner core was AE13^-^, while the middle and outer cores were K14^+^ and AE13^+^, respectively. These results revealed that not only Matrigel but also collagen I, the most ubiquitous extracellular matrix protein in the body, promoted the differentiation of epithelial cells to hair cortex cells in particular combinations (FF-C and AF-C), whereas the cells in FA-M remained their initial core–shell configuration even with Matrigel supplementation.

A previous study showed that hair follicle-bearing skin organoids could be generated from human pluripotent stem cells, although it took ~ 140 days to culture^16^. The hair follicles in the skin organoid exhibited various tissue-specific structures, such as IRS, DP, dermal sheath, ORS, hair matrix, hair bulge, and pigmented hair shafts. In this study, the hair-peg-like sprouting elongated up to ~ 1 mm after 10 days of culture. However, they lacked several in vivo hair follicle structures. Hence, further investigations will be necessary to find missing pieces for the maturation of hair follicles. Nonetheless, we believe this simple process and short culture period are suitable for the high-throughput screening of drug candidates, so we investigated its utility in hair growth-promoting drugs.

### Responses of hair peg-like sprouting to hair growth-promoting drugs

**Figure 4:**
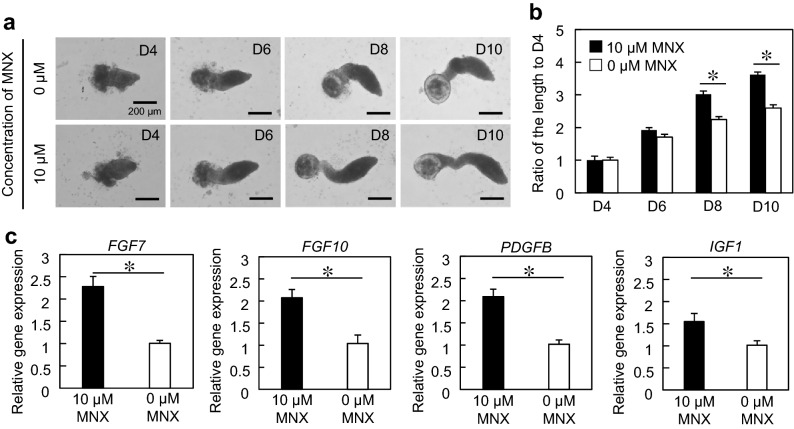
Hair growth-promoting drug testing on hair follicloids. (**a**) Microscope images of hair follicloids cultured with/without minoxidil (MNX) for 10 days. Hair follicloids were permeabilized and observed using a stereomicroscope. (**b**) The length of sprouting structures with/without MNX. The graph shows the ratio of the length on days six, eight, and ten compared to that on day four. Numerical variables were statistically evaluated using Student’s *t*-test; * indicates *p* < 0.05. (**c**) Relative expression of hair growth-associated genes. *GAPDH* was used as a reference gene to normalize expression. Error bars represent the standard error of the mean calculated from three experiments for each condition. Numerical variables were statistically evaluated using Student’s *t*-test; * indicates *p* < 0.05.

Minoxidil (MNX) prolongs the hair growth phase by stimulating EMIs^24^. The cells in AA-M were cultured for 4 days and then exposed to a culture medium supplemented with 10 μM MNX for the following 6 days of culture. Hair peg-like sprouting was significantly longer in the MNX-treated group than in the non-treated control group at eight and 10 days of culture (Fig. 4a, b). We further investigated the expression of hair growth-associated genes, including fibroblast growth factor (*FGF*) 7, *FGF10*, platelet-derived growth factor-beta (*PDGFB*), and insulin-like growth factor 1 (*IGF1*). The expression of these genes significantly increased upon treatment with MNX (Fig. 4c). During the in vivo anagen phase, these genes were strongly upregulated in DP cells, confirming the stimulation of epithelial cell proliferation via EMIs ^25–27^. Our experimental data are consistent with the events observed in vivo. MNX was initially developed as an antihypertensive agent, and its effect on increasing blood flow has been suggested as a mechanism for accelerating hair growth. Other mechanisms have since been proposed, including potassium channel opening^28^, β-catenin signaling activation^29^, extracellular signal-regulated kinase and Akt signaling activation^30^, and increased release of growth factors such as FGF7 and IGF1^31,32^. In the present study, MNX treatment upregulated the expression of growth factors, as shown in Fig. 4c. Taken together, the results obtained in this study suggest that hair peg-like sprouting can be an indicator of drug efficacy and our approach might be useful for elaborating mechanisms of drug action.

### Hair peg-like sprouting using hair follicle-derived cells from a patient with androgenetic alopecia (AGA)

**Figure 5:**
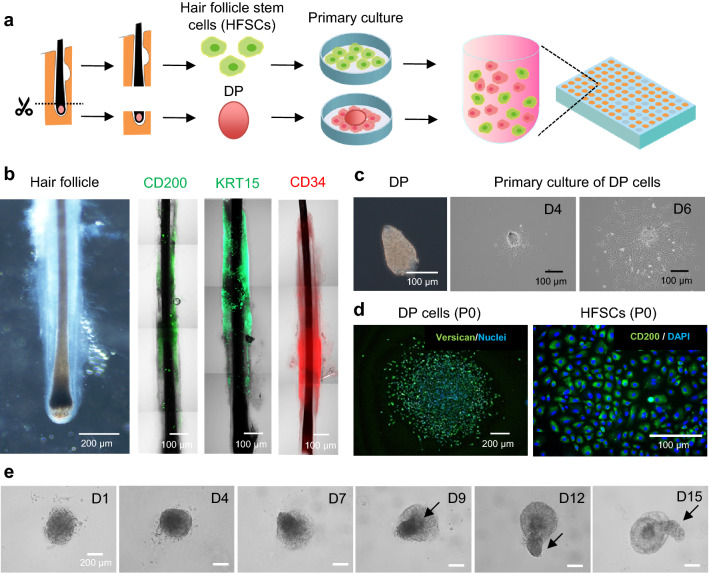
Preparation of hair follicloids using patient hair follicle-derived cells. (**a**) Preparation of hair follicle stem cells (HFSCs) and dermal papilla (DP) cells from hair follicles of patients with androgenic alopecia. Dissociated HFSCs and DP tissues were seeded on the dishes for primary culture. Proliferated HFSCs and DP cells were suspended in culture media containing Matrigel and cultured in a 96-well plate. (**b**) Native human hair follicles. Microscope and immunostaining images were taken before cell dissociation. (**c**) DP cell culture. Isolated DPs adhered to and proliferated on the culture dishes. (**d**) Expression of stem cell markers in HFSC and DP cells. Primary cultures containing HFSCs and DP cells were visualized through the immunohistochemical staining of versican and CD200. (**e**) Time-course of sprouting of hair follicloids.

We further investigated whether our approach applies to cells from the hair follicles of a patient with AGA (Fig. 5a). Since a hair growth-promoting drug should be effective in patients with AGA, it is important to test it on them at the early stage of drug development. The hair follicle contains two types of stem cells, hair follicle stem cells (HFSCs) and DP cells. HFSCs are epithelial cells that reside in the middle of the ORS and are identified as CD200^+^, K15^+^, and CD34^-^ cells ^33,34^. We first confirmed the presence of HFSCs in the ORS of AGA patient-derived hair follicles using immunostaining (Fig. 5b). On the other hand, DP cells are mesenchymal cells that reside in the hair bulb. To isolate cells with epithelial and mesenchymal lineage, hair bulbs containing DP cells were initially surgically dissected and cultured in a cell culture dish (Fig. 5c, d). The remaining portions of the hair follicles were enzymatically treated to dissociate epithelial cells, including HFSCs, and cultured in an iMatrix-511-coated dish. Then, the two cell types were mixed in a medium supplemented with Matrigel and cultured in the same manner as the healthy hair follicle-derived cells. Hair peg-like sprouting was observed in the AGA patient-derived cells (Fig. 5e). Although the sprouting length was shorter than that in AA-M, spontaneous aggregation, spatial separation of the two cell types inside the aggregate, and subsequent elongation of the epithelial cell aggregate portion were observed. Their shorter length could be attributed to their AGA origin. Another possible cause is that, unlike commercially available cells from healthy hair follicles, cells of AGA origin were immediately used for the experiments in this study after the cell isolation procedures. Enzyme-induced damage during isolation from tissues might have affected cell behaviors.

Conventional organ culture methods using freshly isolated hair follicles have been used to examine the effects of hair drugs, including MNX^35^. However, using them to test many drug candidates is challenging since only a limited number of hair follicles are available. In this aspect, our approach may provide a better platform. Many test tissues can be prepared from a few hair follicles because the number of cells can be increased upon cell culture passage. Indeed, cells from healthy donors were passaged at least twice in the lab prior to the hair peg-like sprouting assay in this study.

Human epithelial and mesenchymal cells (even commercially available healthy donor-derived cells and freshly isolated AGA donor-derived cells) have yet to generate mature hair follicles and hair shafts, as previously observed in murine embryonic cells^18,36^. Many possible factors in both cell expansion culture and subsequent aggregation culture need to be investigated to address this issue. In the cell expansion culture, epithelial and mesenchymal cells gradually lose their hair regenerative ability^37,38^. To maintain the hair growth-inducing ability of DP cells in culture, we have found several approaches, such as using a PI3k/Akt signaling pathway activator^39^ and electrical stimulation^40^. Cues for the essential components of hair follicle maturation may be found during hair follicle morphogenesis in vivo. Hair follicle development and postnatal hair cycles in vivo are governed by the microenvironment surrounding the hair follicles, such as different types of cells and extracellular matrices^41,42^. The upregulation of trichogenic gene expression in epithelial and mesenchymal cell aggregates was shown upon co-culture with neighboring cells, such as vascular endothelial cells and adipose-derived stem cells, resulting in superior hair regeneration efficiency upon subcutaneous transplantation^43,44^. Adding other types of cells and extracellular matrices to this approach will be the focus of our subsequent studies.

## Conclusion

Hair peg-like sprouting was generated at very high frequencies (~ 100%) within 10 days of culture using combinations of fetal epithelial cells/fetal mesenchymal cells/Matrigel, fetal epithelial cells/fetal mesenchymal cells/collagen, and adult epithelial cells/adult mesenchymal cells/Matrigel. Hair peg-like sprouting in adult epithelial cells/adult mesenchymal cells/Matrigel elongated more when exposed to minoxidil compared with no exposure, suggesting the feasibility of this approach for hair growth-promoting drug testing. Hair peg-like sprouting was also observed in AGA patient hair follicle-derived cells, but the sprouting length was shorter than that with healthy donors. In addition, none of the cells and extracellular matrix combinations used in this study resulted in the formation of mature hair follicles, as previously reported with the fetal mouse skin-derived cells. Further improvement of this approach to generate mature hair follicles will open up new avenues for finding hair growth-promoting drugs for the treatment of alopecia and in improving hair pigmentation since the production and transport of melanosomes can be continuously monitored under the microscope using this in vitro culture system^18^. Furthermore, mature hair follicles may be available for tissue grafts of surgical hair transplantation as a novel approach to hair regenerative medicine.

## Methods

### Preparation of human epithelial and mesenchymal cells

Human fetal epidermal keratinocytes (fetal Epi; ScienCell Research Laboratories, Carlsbad, CA, USA), human adult follicular keratinocytes (adult Epi; ScienCell Research Laboratories), human fetal dermal fibroblasts (fetal Mes; ScienCell Research Laboratories), and human adult DP cells (adult Mes; PromoCell, Heidelberg, Germany) were used in this study. Cells from passages up to 2–3 were used for the organoid culture.

### Preparation of patient-derived HFSCs and DP cells

After obtaining informed consent, human scalp hair follicles were obtained from patients with androgenic alopecia. This study was approved by the ethics committee of the Kanagawa Institute of Industrial Science and Technology (approval number: S-2019-01) and Yokohama National University (approval number: 2021-04). The study was performed in accordance with their guidelines and the Declaration of Helsinki.

Hair bulbs containing DP cells and dermal sheath cups were dissected from the hair follicles and treated with 4.8 U/mL dispase II and 100 U/mL collagenase in a 1:1 solution mixture of Hank’s balanced salt solution/phosphate-buffered saline (HBSS/PBS) for 30 min at 37 °C. After centrifugation (180 × *g*, 3 min), dissociated DPs were seeded on a cell culture plate and maintained in a follicle DP cell growth medium (PromoCell), which was replaced with fresh medium after every 2 to 3 days.

The remaining hair follicle tissues were treated with 4.8 U/mL dispase II and 100 U/mL collagenase in an HBSS/PBS (1:1) solution for 10 min at 37 °C. The collagen sheath was surgically removed and treated with 0.05% trypsin in PBS for 60 min at 37 °C. The debris and undissociated tissues were removed using a cell strainer. After centrifugation (180 × *g*, 3 min), the human HFSCs were seeded in an iMatrix 511-coated dish and cultured in a StemFit AK02N medium (Ajinomoto, Tokyo, Japan) supplemented with 10 µM CultureSure® Y-27632 and 5 µM CultureSure® A83-01 solutions (FUJIFILM Wako Pure Chemical Corporation, Osaka, Japan). The spent medium was replaced with fresh medium after every 2–3 days.

### Preparation of human hair follicle growth model

#### Using fetal/adult Epi and Mes cells

To engineer hair follicloids, fetal/adult Epi (5 × 10^3^ cells) and Mes (5 × 10^3^ cells) cells were suspended in 0.2 mL advanced Dulbecco's modified eagle medium/Nutrient mixture F-12 (DMEM/F-12) containing 2% v/v Matrigel (Corning, Glendale, Arizona, USA) or 2% v/v collagen gel (Cellmatrix type I-A; Nitta Gelatin, Osaka, Japan) and seeded into the wells of a non-cell adhesive, round-bottom, 96-well plate (Prime Surface 96U plate, Sumitomo Bakelite, Akita, Japan). The plates were centrifuged at 100 × *g* for 2 min, cooled at 4 °C in a refrigerator (MPR-S313-PJ, PHC, Japan) for at least 30 min, and incubated in a 5% CO_2_ incubator (MCO-170AICUVD, PHC, Japan) at 37 °C. Cells were maintained in DMEM/F-12 medium, where 0.1 mL of the spent medium was replaced with the same amount of fresh medium in each well after every 2 days. Hair sprouts from hair follicloids were characterized using an all-in-one fluorescence microscope (BZ-X810, Keyence, Osaka, Japan), and these structures were further analyzed via histological and immunohistochemical staining. The relative expression levels of genes associated with hair morphogenesis were evaluated using real-time reverse transcription-polymerase chain reaction (RT-PCR).

#### Using AGA patient-derived HFSCs and DP cells

After primary culture, most of the DP cells and HFSCs expressed stem cell markers, including versican and CD200, respectively (Fig. 5d). These cells were mixed at a 1:1 ratio and cultured in DMEM/F-12 medium supplemented with 2% v/v Matrigel. Cells initially formed a single aggregate and spontaneously formed a hair-like sprouting structure after 9 days, which grew up to 15 days of culture (Fig. 5e). These hair-like sprouts were characterized using an all-in-one fluorescence microscope (BZ-X810, Keyence).

### In vitro hair-like sprouting assay

To investigate the effect of hair growth-promoting drugs on the growth of generated hair sprouts, adult Epi (5 × 10^3^ cells) and Mes (5 × 10^3^ cells) cells were suspended in 0.2 mL DMEM/F-12 containing 2% v/v Matrigel and seeded into the wells of a Prime Surface 96U plate. DMEM/F-12 medium was supplemented with 10 μM MNX (Merck Millipore, Burlington, MA, USA) from days four to ten after seeding. Then, 0.1 mL of the spent medium was replaced with the same amount of fresh medium after every 2 days. The length of hair sprouts was observed using an all-in-one fluorescence microscope (BZ-X810, Keyence) after 10 days.

### Histological staining

The samples were washed with PBS, fixed with 4% formaldehyde overnight at 25 °C, rinsed three times with PBS, and successively submerged into 10%, 20%, and 30% sucrose solutions (FUJIFILM Wako Laboratory Chemicals) for 1 h. Next, the samples were transferred to a Tissue-Tek® cryomold (Sakura Finetek, Tokyo, Japan). The sucrose solution was carefully aspirated to ensure that only spheroids would remain, and the Tissue-Tek® cryomold was filled with Tissue-Tek O.C.T. compound (Sakura Finetek). After embedding the samples in the O.C.T. compound, they were dissected into 10-μm-thick sections and stained using Meyer’s hematoxylin and eosin Y (Muto Pure Chemicals, Tokyo, Japan). An all-in-one fluorescence microscope (BZ-X810, Keyence) was used for imaging.

### Immunohistochemical staining

The human hair follicloids were first blocked in PBS containing 3% goat serum (Abcam, Cambridge, CB2 0AX, UK) and 0.3% Triton X-100 (Sigma Aldrich, St. Louis, MO, USA) for 1 h at 25 °C and subsequently incubated overnight with anti-cytokeratin 14 SP53 (ab119695; Abcam), anti-K40 AE13 (ab16113; Abcam), and anti-versican (ab19345, Abcam) antibodies at 4 °C. The samples were incubated with the corresponding secondary antibodies (Alexa Fluor® 488 ab150077; Abcam) suspended in the blocking solution for 2 h at 25 °C and finally stained with 4′,6-diamidino-2-phenylindole in PBS for 10 min. Next, 10-μm-thick frozen sections were prepared using the same steps as histological staining. An all-in-one fluorescence microscope (BZ-X810, Keyence) was used for fluorescence imaging.

### Gene expression analysis using RT-PCR

Total RNA was extracted from the samples using an RNeasy mini kit (QIAGEN; Germantown, MD, US), and cDNA was synthesized via reverse transcription using an RT^2^ First strand kit (QIAGEN) or ReverTra Ace™ qPCR RT Kit (Toyobo, Osaka, Honshu, Japan), according to the manufacturer’s instructions. We performed qPCR using a StepOnePlus™ Real-Time PCR system (Applied Biosystems) and TB Green® Premix Ex Taq II reagent (Takara Bio). We used the following primers to assess the expression levels of the respective genes: *FGF7* (forward 5′-TTGTGGCAATCAAAGGGGTG-3′ and reverse 5′-CCTCCGTTGTGTGTCCATTT-3′), *FGF10* (forward 5′-TTCAAGGAGATGTCCGCT-3′ and reverse 5′-GATGCTGTACGGGCAGTT-3′), *PDGFB* (forward 5′-GAAGGAGCCTGGGTTCCC-3′ and reverse 5′-TTTCTCACCTGGACAGGT-3′), *IGF1* (forward 5′-TTCAACAAGCCCACAGGG-3′ and reverse 5′-GGTGCGCAATACATCTCC-3′), and *GAPDH* (forward 5′-TGGAAATCCCATCACCATCTTC-3′ and reverse 5′-CGCCCCACTTGATTTTGG-3′). The expression levels of all the genes were normalized to that of *GAPDH*. Relative gene expression levels were determined using the 2^−ΔΔCt^ method.

### Statistical analysis

Statistical analyses of the lengths of the sprouting structures and the generation efficiency of sprouting structures were conducted using Tukey's test. Statistical analyses of the gene expression levels were conducted using Student’s *t*-test, and the results were considered statistically significant at *p* < 0.05. We cultured at least 24 hair follicloids per treatment condition, which were used to calculate sprouting length. All data are presented as the mean ± standard error.

## Supplementary Information

Supplementary Information.

## Data Availability

All data generated or analyzed during this study are included in the main text of this manuscript.

## References

[1] Phillips TG, Slomiany WP, Allison R (2017). Hair loss: common causes and treatment. Am. Fam. Physician.

[2] Lin RL, Garibyan L, Kimball AB, Drake LA (2016). Systemic causes of hair loss. Ann. Med..

[3] Santos Z, Avci P, Hamblin MR (2015). Drug discovery for alopecia: gone today, hair tomorrow. Expert Opin. Drug Discov..

[4] Ghanemi A (2015). Cell cultures in drug development: applications, challenges and limitations. Saudi Pharm. J..

[5] Znidaric M, Zurga ZM, Maver U (2021). Design of in vitro hair follicles for different applications in the treatment of alopecia-a review. Biomedicines.

[6] Ji S, Zhu Z, Sun X, Fu X (2021). Functional hair follicle regeneration: an updated review. Signal. Transduct. Target Ther..

[7] Driskell RR, Clavel C, Rendl M, Watt FM (2011). Hair follicle dermal papilla cells at a glance. J. Cell Sci..

[8] Bejaoui M, Oliva AK, Ke MS, Ferdousi F, Isoda H (2022). 3D spheroid human dermal papilla cell as an effective model for the screening of hair growth promoting compounds: examples of minoxidil and 3,4,5-tri-O-caffeoylquinic acid (TCQA). Cells.

[9] Millar SE (2002). Molecular mechanisms regulating hair follicle development. J. Invest. Dermatol..

[10] Sennett R, Rendl M (2012). Mesenchymal-epithelial interactions during hair follicle morphogenesis and cycling. Semin. Cell Dev. Biol..

[11] Toyoshima K-e (2012). Fully functional hair follicle regeneration through the rearrangement of stem cells and their niches. Nat. Commun..

[12] Kageyama T (2018). Spontaneous hair follicle germ (HFG) formation *in vitro*, enabling the large-scale production of HFGs for regenerative medicine. Biomaterials.

[13] Kageyama T, Yan L, Shimizu A, Maruo S, Fukuda J (2019). Preparation of hair beads and hair follicle germs for regenerative medicine. Biomaterials.

[14] Nanmo A (2022). Bioprinting of hair follicle germs for hair regenerative medicine. Acta Biomater..

[15] Abaci HE (2018). Tissue engineering of human hair follicles using a biomimetic developmental approach. Nat. Commun..

[16] Lee JY (2020). Hair-bearing human skin generated entirely from pluripotent stem cells. Nature.

[17] Ma J (2022). Establishment of human pluripotent stem cell-derived skin organoids enabled pathophysiological model of SARS-CoV-2 infection. Adv. Sci. (Weinh).

[18] Kageyama T (2022). Reprogramming of three-dimensional microenvironments for in vitro hair follicle induction. Sci. Adv..

[19] Castro AR, Logarinho E (2020). Tissue engineering strategies for human hair follicle regeneration: How far from a hairy goal?. Stem Cells Transl. Med..

[20] Wu X, Scott L, Washenik K, Stenn K (2014). Full-thickness skin with mature hair follicles generated from tissue culture expanded human cells. Tissue Eng. Part A.

[21] Abreu CM (2021). Rescuing key native traits in cultured dermal papilla cells for human hair regeneration. J. Adv. Res..

[22] Leng X (2020). Dissociated skin cells regenerate hair follicles in a microwound, “The Punch Assay”. Exp. Dermatol..

[23] Su Y (2019). Pre-aggregation of scalp progenitor dermal and epidermal stem cells activates the WNT pathway and promotes hair follicle formation in *in vitro* and *in vivo* systems. Stem Cell Res. Ther..

[24] Messenger AG, Rundegren J (2004). Minoxidil: mechanisms of action on hair growth. Br. J. Dermatol..

[25] Kawano M (2005). Comprehensive analysis of FGF and FGFR expression in skin: FGF18 is highly expressed in hair follicles and capable of inducing anagen from telogen stage hair follicles. J. Invest. Dermatol..

[26] Tomita Y, Akiyama M, Shimizu H (2006). PDGF isoforms induce and maintain anagen phase of murine hair follicles. J. Dermatol. Sci..

[27] Greco V (2009). A two-step mechanism for stem cell activation during hair regeneration. Cell Stem Cell.

[28] Rossi A (2012). Minoxidil use in dermatology, side effects and recent patents. Recent Pat. Inflamm. Allergy Drug Discov..

[29] Kwack MH, Kang BM, Kim MK, Kim JC, Sung YK (2011). Minoxidil activates β-catenin pathway in human dermal papilla cells: a possible explanation for its anagen prolongation effect. J. Dermatol. Sci..

[30] Han JH (2004). Effect of minoxidil on proliferation and apoptosis in dermal papilla cells of human hair follicle. J. Dermatol. Sci..

[31] Lee CY (2018). Hair growth is promoted by BeauTop via expression of EGF and FGF-7. Mol. Med. Rep..

[32] Sattur SS, Sattur IS (2021). Pharmacological Management of Pattern Hair Loss. Indian J. Plast. Surg..

[33] Inoue K (2009). Differential expression of stem-cell-associated markers in human hair follicle epithelial cells. Lab. Invest..

[34] Ohyama M (2006). Characterization and isolation of stem cell-enriched human hair follicle bulge cells. J. Clin. Investig..

[35] Buhl AE, Waldon DJ, Kawabe TT, Holland JM (1989). Minoxidil stimulates mouse vibrissae follicles in organ culture. J. Invest. Dermatol..

[36] Kageyama T, Anakama R, Togashi H, Fukuda J (2022). Impacts of manipulating cell sorting on *in vitro* hair follicle regeneration. J. Biosci. Bioeng..

[37] Higgins CA, Chen JC, Cerise JE, Jahoda CA, Christiano AM (2013). Microenvironmental reprogramming by three-dimensional culture enables dermal papilla cells to induce *de novo* human hair-follicle growth. Proc. Natl. Acad. Sci. U S A.

[38] Sanchez-Danes A, Blanpain C (2017). Maintaining hair follicle stem cell identity in a dish. EMBO J..

[39] Yamane M (2022). Effects of the PI3K/Akt signaling pathway on the hair inductivity of human dermal papilla cells in hair beads. J. Biosci. Bioeng..

[40] Yan L (2022). Electrical stimulation to human dermal papilla cells for hair regenerative medicine. J. Biosci. Bioeng..

[41] Chen CL, Huang WY, Wang EHC, Tai KY, Lin SJ (2020). Functional complexity of hair follicle stem cell niche and therapeutic targeting of niche dysfunction for hair regeneration. J. Biomed. Sci..

[42] Jahoda CAB, Christiano AM (2011). Niche crosstalk: intercellular signals at the hair follicle. Cell.

[43] Kageyama T, Chun Y-S, Fukuda J (2021). Hair follicle germs containing vascular endothelial cells for hair regenerative medicine. Sci. Rep..

[44] Nakajima R, Tate Y, Yan L, Kageyama T, Fukuda J (2021). Impact of adipose-derived stem cells on engineering hair follicle germ-like tissue grafts for hair regenerative medicine. J. Biosci. Bioeng..

