# Supplementary material for: In vitro hair follicle growth model for drug testing
Source: Sci Rep. 2023 Mar 24;13:4847. doi: 10.1038/s41598-023-31842-y (PMC10038375; doi:10.1038/s41598-023-31842-y)
Supplement: Supplementary file 1 — Supplementary Information. [file 41598_2023_31842_MOESM1_ESM.docx]

**Supplementary information**

***In vitro* hair follicle growth model for drug discovery**

Tatsuto Kageyama^1, 2, 3^, Hikaru Miyata^1^, Jieun Seo^1^, Ayaka Nanmo^1^, and Junji Fukuda^1, 2^*

^1^Faculty of Engineering, Yokohama National University, 79-5 Tokiwadai, Hodogaya-ku, Yokohama, Kanagawa 240-8501, Japan.

^2^Kanagawa Institute of Industrial Science and Technology, 3-2-1 Sakado, Takatsu-ku, Kawasaki, Kanagawa 213-0012, Japan.

^3^Japan Science and Technology Agency (JST)-PRESTO, 4-1-8 Honcho, Kawaguchi, Saitama 332-0012, Japan.

**
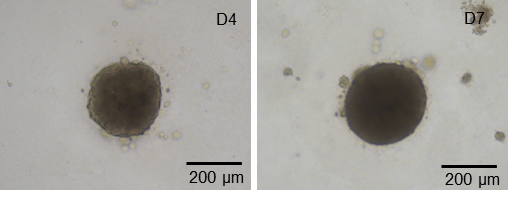
**

**Supplemental Fig. 1. Long-term culture of adult epithelial cell aggregates.** Hair peg-like structures were not formed from spherical cell aggregates.
